# Ultrasound-Guided Ulnar Nerve Hydrodissection for Post-brachioplasty Cubital Tunnel Syndrome in a Patient With Congenital Myasthenic Syndrome: A Case Report

**DOI:** 10.7759/cureus.117239

**Published:** 2026-09-30

**Authors:** Zainab Farooq, Nate Tomilonus, Alex Abouafech, Joe Bhagratie, Stephen Westfall

**Affiliations:** 1 Medicine, Lake Erie College of Osteopathic Medicine, Bradenton, USA; 2 Medicine, Lake Erie College of Osteopathic Medicine, Lakewood Ranch, USA; 3 Orthopedics, Baptist Medical Center Jacksonville, Jacksonville, USA

**Keywords:** brachioplasty, congenital myasthenic syndrome, cubital tunnel syndrome, ulnar nerve entrapment, ultrasound-guided hydrodissection

## Abstract

Cubital tunnel syndrome is a common entrapment neuropathy of the upper extremity, typically resulting from compression of the ulnar nerve at the elbow. Postsurgical scarring is a less frequently discussed etiology, particularly following cosmetic procedures such as brachioplasty. We present the case of a 31‑year‑old female with a history of massive weight loss and bilateral brachioplasty who developed progressive ulnar neuropathic symptoms despite normal electrodiagnostic testing. The patient also carried a diagnosis of congenital myasthenic syndrome (CMS), complicating clinical interpretation. Ultrasound demonstrated ulnar nerve enlargement and tethering beneath brachioplasty scar tissue. The patient was successfully treated with ultrasound‑guided ulnar nerve hydrodissection using dextrose, local anesthetic, and corticosteroid. This case highlights the limitations of electromyography (EMG) in dynamic or scar‑related neuropathies, the potential contribution of brachioplasty scarring to cubital tunnel syndrome, and the role of ultrasound‑guided hydrodissection as a minimally invasive alternative to surgery.

## Introduction

Congenital myasthenic syndrome (CMS) refers to a rare heterogeneous group of hereditary disorders characterized by fatigue and muscle weakness due to impairment in neuromuscular transmission. The most common initial symptoms include weakness of extremities, ptosis, proximal muscle weakness, clumsy gait, difficulty eating solid foods, and recurrent respiratory tract infections [1]. Cubital tunnel syndrome is the second most common peripheral neuropathy in the upper limb. It occurs due to ulnar nerve compression within the fibro-osseous cubital tunnel at the elbow joint [2]. Normally, with elbow range of motion, the ulnar nerve is subjected to compression, traction, and frictional forces. As the elbow is flexed, the arcuate ligament elongates, producing a decrease in canal volume of 55%. Cubital tunnel syndrome may develop because of various factors, including repetitive elbow motion, prolonged elbow flexion, or direct compression [3]. Less commonly, iatrogenic causes such as postoperative scarring and extraneural fibrosis are possible causes of recurrent peripheral nerve problems and may lead to nerve tethering and irritation [4]. Diagnosis is typically made with electrophysiology. However, ultrasound had higher sensitivity than electrophysiology in clinically very mild (20% and 3% for ultrasound and electrophysiology, respectively) and mild (62% and 47% for ultrasound and electrophysiology, respectively) neuropathies, had greater localizing ability in axonal ulnar neuropathies, and identified nerve hypermobility [5].

Brachioplasty, or upper arm lift, is a body-contouring procedure designed to remove excess skin and subcutaneous tissue from the upper arm, typically extending from the axilla to the elbow. This procedure is most commonly indicated in patients who have experienced massive weight loss, aging-related dermal laxity, or localized adiposity unresponsive to liposuction [6]. Brachioplasty involves extensive medial arm dissection and carries a known risk of sensory nerve injury and problematic scarring. Regarding sensory nerve injury, the literature documents nerve-related complications occurring in 2.47% of brachioplasty cases (95% CI, 1.45-4.18%) in a meta-analysis of 1,578 patients [7].

Ultrasound‑guided nerve hydrodissection has emerged as a treatment option for entrapment neuropathies, particularly when symptoms are predominantly sensory and electrodiagnostic studies are normal. Ultrasound-guided nerve hydrodissection is a technique whereby fluid is used to separate a nerve from surrounding tissue as a means to alleviate symptoms of peripheral entrapment neuropathies. Possible mechanisms for improvement in symptoms include improvement in the function of the nervi nervorum and vasa nervorum [8]. This case is notable for the coexistence of CMS, a neuromuscular junction disorder that may confound symptom interpretation and heighten sensitivity to peripheral nerve irritation.

## Case presentation

A 31-year-old female presented with progressive left medial elbow pain, numbness, and burning paresthesia radiating into the ulnar forearm and fifth digit. She had a history of massive weight loss of approximately 160-170 lb and underwent bilateral brachioplasty two years prior. Her medical history was notable for CMS, which was reportedly well controlled without regular neurology follow-up. Following the brachioplasty, she experienced intermittent abnormal sensations around the left elbow that became persistent and progressively worse over the preceding three to four weeks. Symptoms included burning pain, pins-and-needles sensation, and numbness along the medial arm and small finger, most pronounced in the afternoon. She denied weakness, object dropping, or neck pain. Conservative management with nonsteroidal anti-inflammatory drugs, muscle relaxants, topical patches, and gabapentin 100 mg nightly did not provide relief.

On physical examination, a well-healed medial arm brachioplasty scar was noted, as shown in Figure 1. Palpation revealed tenderness over the medial epicondyle and cubital tunnel. Neurologic examination demonstrated a positive Tinel’s sign at the left cubital tunnel without intrinsic hand weakness, though there was sensory disturbance distal to the elbow. Elbow range of motion was full and stable. Lateral and anteroposterior (AP) radiographs of the left elbow were normal, as shown in Figure 2. Electrodiagnostic testing of both upper extremities showed no evidence of ulnar mononeuropathy, cervical radiculopathy, or brachial plexopathy. However, ultrasound demonstrated an enlarged ulnar nerve at the cubital tunnel with sonographic tenderness and tethering deep to the brachioplasty scar tissue, as shown in Figure 3.

**Figure 1:**
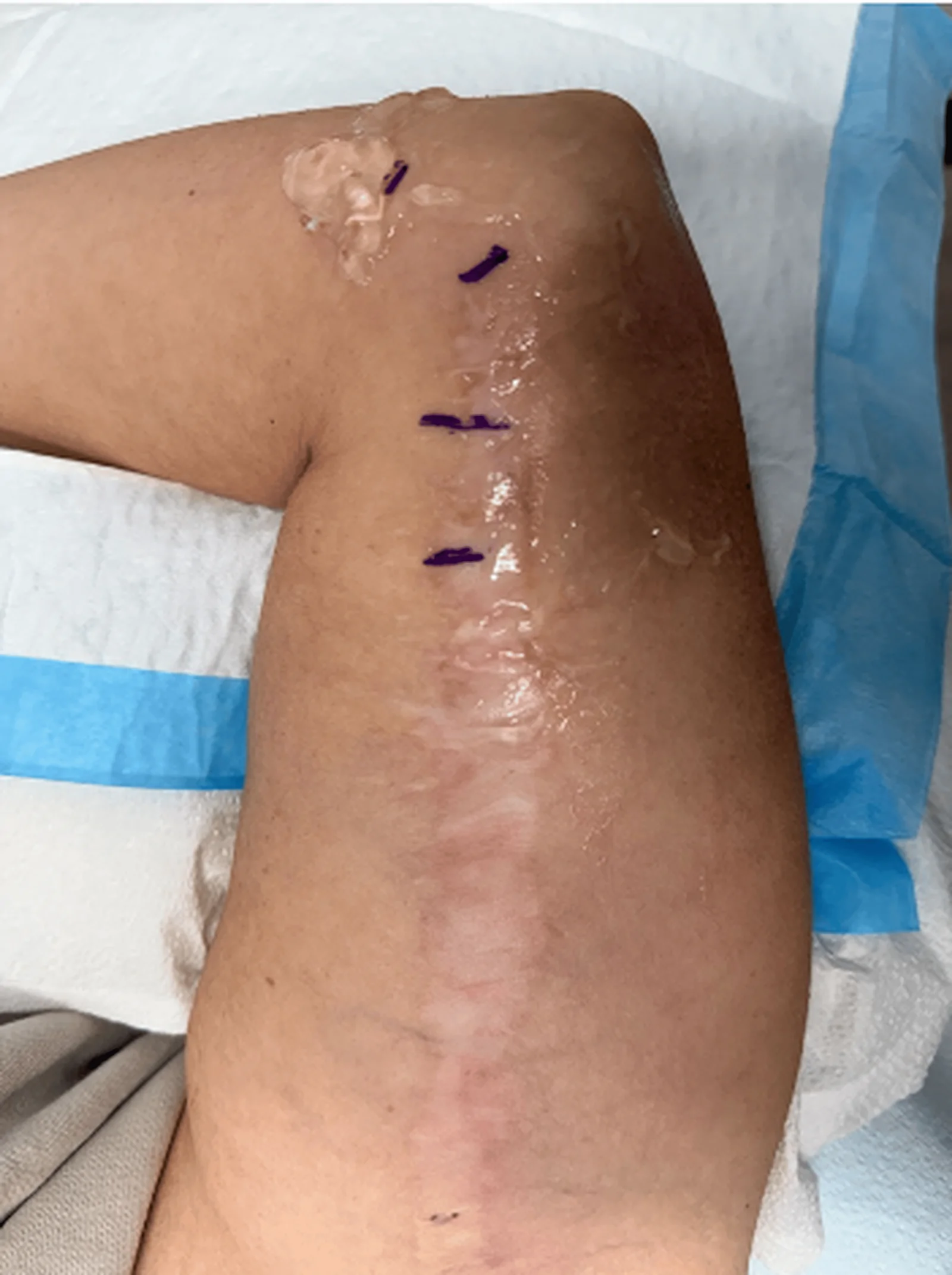
A well-healed left medial arm brachioplasty with scarring marked for ultrasound-guided nerve hydrodissection

**Figure 2:**
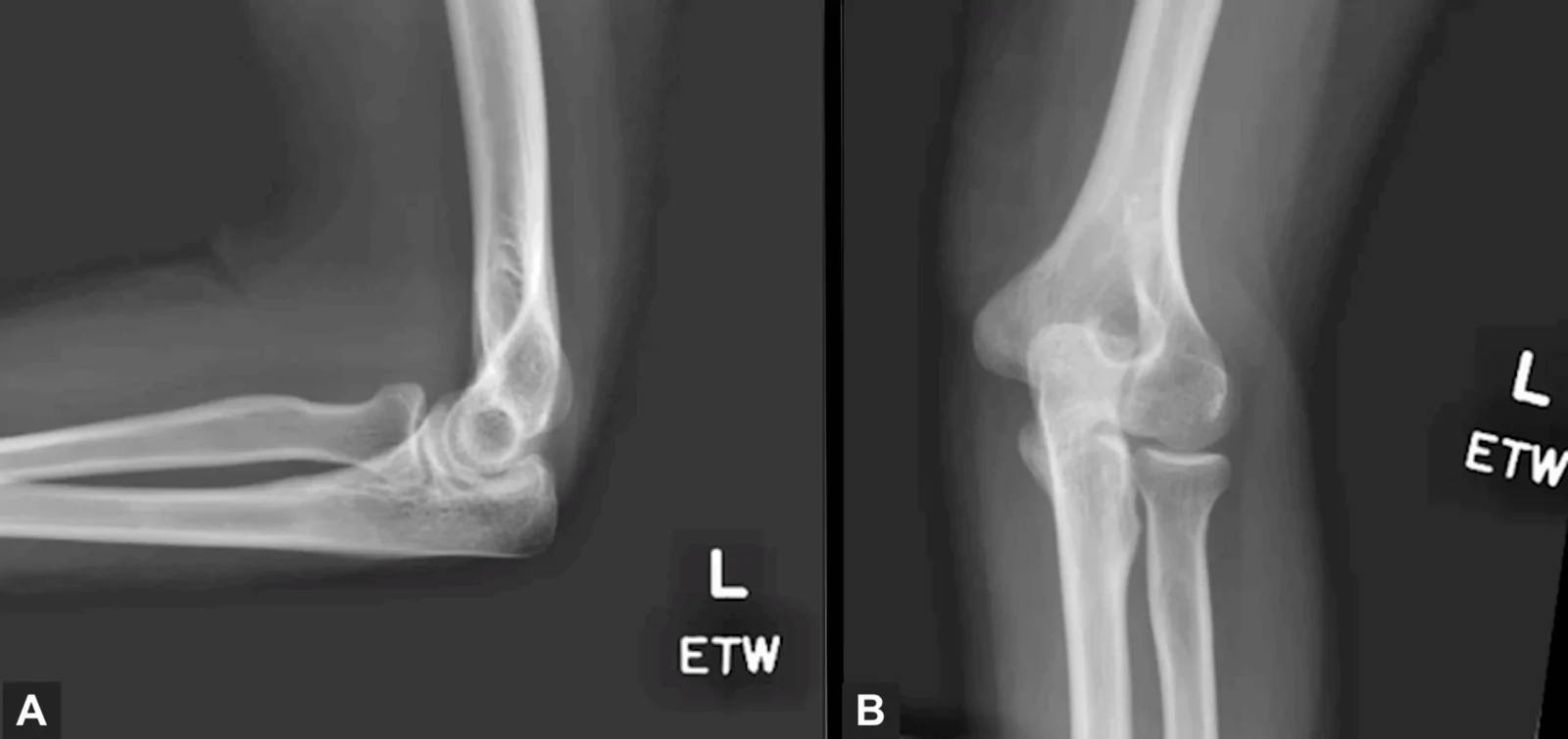
Normal lateral (A) and anteroposterior (B) radiographs of the left elbow

**Figure 3:**
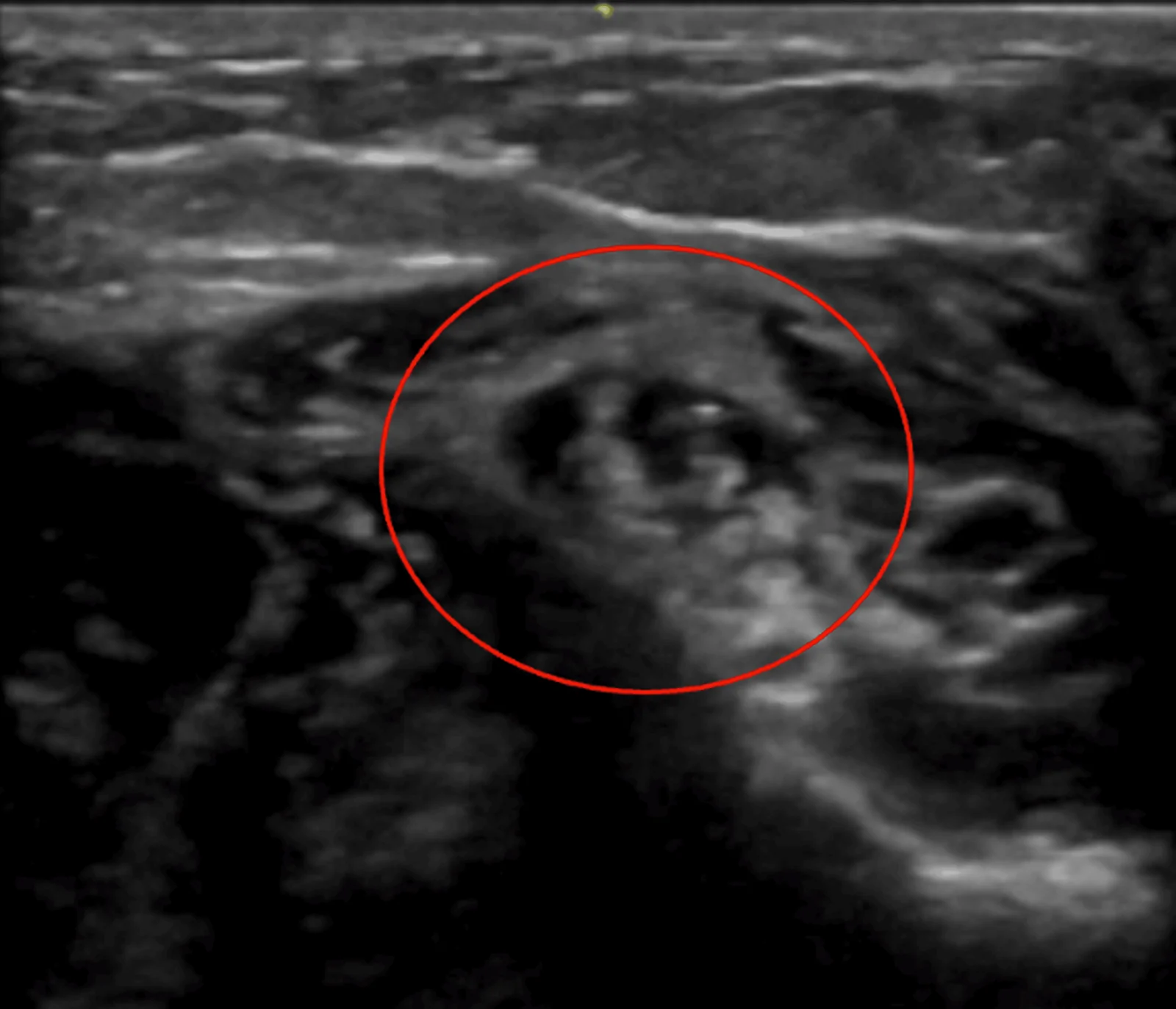
Ultrasound demonstrating an enlarged ulnar nerve at the cubital tunnel, as indicated by the red circle, with sonographic tenderness and tethering deep to the brachioplasty scar tissue

The patient was diagnosed with left cubital tunnel syndrome secondary to postsurgical scarring. She underwent ultrasound-guided ulnar nerve hydrodissection under sterile technique and real-time ultrasound guidance, as shown in Figure 4. The ulnar nerve was identified and circumferentially released from surrounding scar tissue and fascia using a mixture of 5% dextrose in saline, 1% lidocaine, and triamcinolone, with a total volume of 20 mL injected across four sites proximal and distal to the elbow crease. Post-procedure ultrasound confirmed improved nerve mobility and separation from adjacent tissues. The patient tolerated the procedure well and reported immediate pain relief. She was instructed to begin physical therapy with nerve-gliding exercises, modify activities, and consider repeat hydrodissection without corticosteroid if symptoms recurred, with surgical referral reserved for refractory cases.

**Figure 4:**
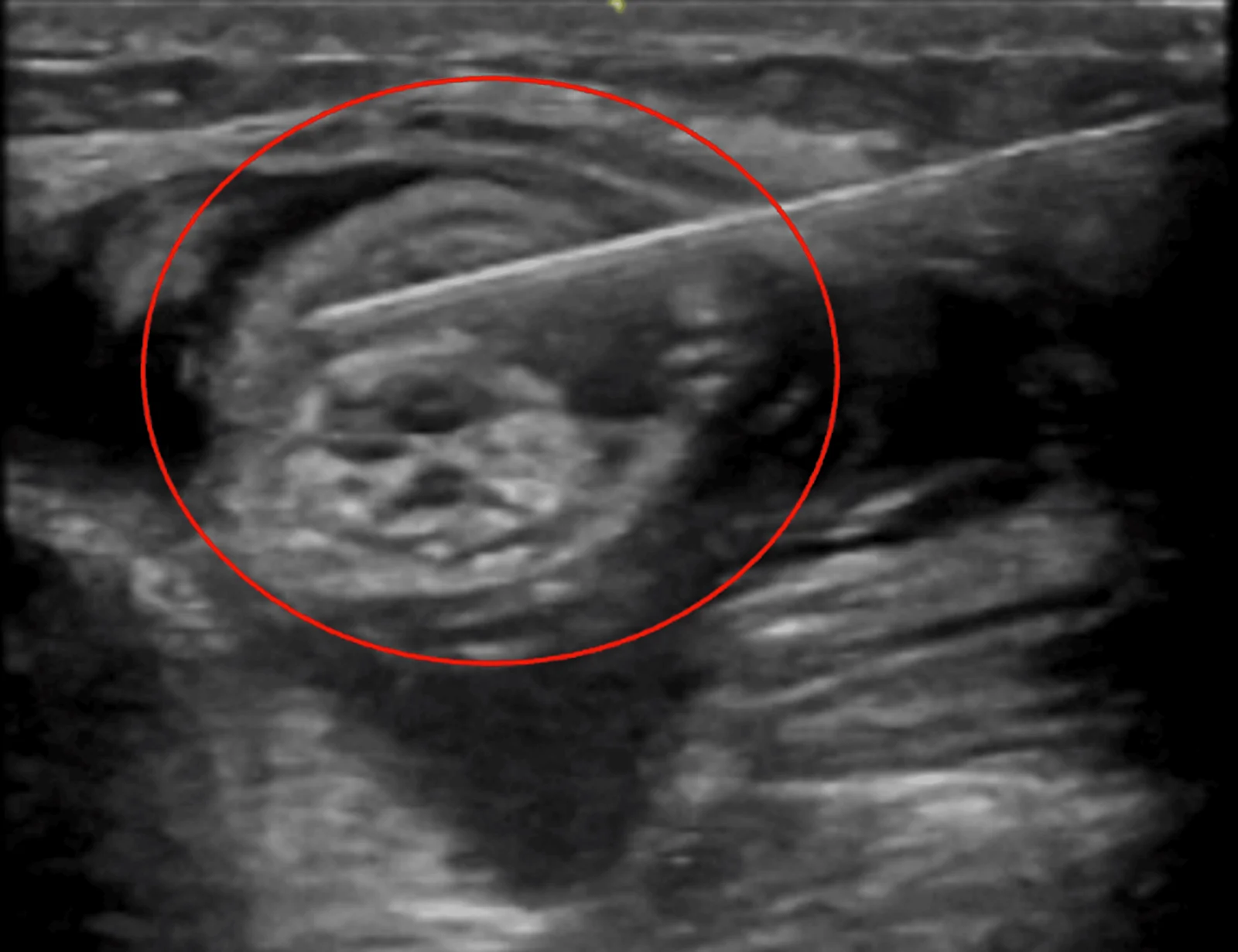
Ultrasound-guided ulnar nerve hydrodissection under sterile technique showing the needle inserting the solution around the nerve and releasing it from scar tissue, as labeled by the red circle

## Discussion

Brachioplasty is associated with a relatively high incidence of sensory complications, particularly involving the ulnar nerve. Extensive dissection along the medial arm can lead to hypertrophic scarring, fascial thickening, and nerve tethering. Hypertrophic scarring is extensively documented, occurring in 24.0% of brachioplasty patients in one series, with aberrant scarring representing 36.4% of all revision procedures [9]. Although cosmetic outcomes are often prioritized, long‑term neurologic sequelae such as delayed‑onset neuropathic pain may occur [10]. Scarring in the region of the cubital tunnel may exacerbate nerve strain during elbow flexion, producing symptoms despite preserved nerve conduction [10].

Electrodiagnostic studies may be normal in early, intermittent, or purely sensory neuropathies. Normal findings on electrodiagnostic studies significantly decrease the likelihood of peripheral neuropathy but are more accurate in the larger, axonal fibers, indicating limitations in detecting early or small fiber pathology [11]. In cases of dynamic compression or scar‑related tethering, electromyography (EMG) may fail to capture pathology, emphasizing the importance of clinical examination and ultrasound evaluation.

CMS affects neuromuscular transmission and may increase patient sensitivity to neuromuscular stressors [12]. Although CMS does not directly cause focal compressive neuropathy, baseline neuromuscular fatigue and altered motor unit recruitment may amplify symptom perception or delay recovery. Additionally, clinicians may be hesitant to pursue surgical intervention in CMS patients due to concerns regarding postoperative weakness [12].

Surgical cubital tunnel release remains the standard for severe or progressive motor deficits. However, surgery carries risks, including infection, nerve injury, prolonged recovery, and scar recurrence in patients with prior arm surgery [13]. Ultrasound‑guided hydrodissection offers several advantages, as it is minimally invasive, performed under local anesthesia, targets scar tethering, and allows real-time visualization of nerve release [8]. Emerging evidence suggests hydrodissection can improve pain and function in mild‑to‑moderate cubital tunnel syndrome, especially when EMG findings are negative and symptoms are sensory‑predominant [8].

## Conclusions

This case illustrates a rare but clinically significant cause of cubital tunnel syndrome following brachioplasty. It underscores the value of ultrasound in identifying nerve entrapment not detected by EMG and supports ultrasound‑guided ulnar nerve hydrodissection as an effective, low‑risk alternative to surgery. Consideration of underlying neuromuscular conditions such as CMS is essential in tailoring treatment and counseling patients regarding prognosis.
